# Event and Comment

**Published:** 1922-11

**Authors:** 


### DENTAL REGISTER

THE MAGAZINE OF DENTISTRY

Vol. LXXVI NOVEMBER, 1922 No. 11

### EVENT AND COMMENT

**A Children's Health Drive** We have discussed extensively and advocated many methods for preventing the universal destruction of the human teeth by dental caries, but so far without satisfactory results.

It sometimes seems to us a hopeless situation, and we fear we shall never make any progress by our present endeavors unless we increase our research more determinedly, or adopt a new line of attack. A new method or a radical modification of the best results we know might help to clear the atmosphere and start a renewed interest in the most logical thing we know; such, for instance, as an endeavor to apply our best known methods to a new field, or one that has not been overworked already. At the present time oral hygiene and prophylaxis seem to have the most claims to the profession and the intelligent laymen. It would seem that there is little that can be said further than to reiterate well-known practices and theories.

We have often wondered whether we are beginning our hygiene and prophylactic ministrations at the proper place in the history of the child to get the best results for health. The ideal time for instituting hygiene seems to be the school age. Children of school age to about eighteen years seem to develop dental caries more readily than at any other period. Whether there is a reason for this or merely traditional, it would be difficult to determine. It seems that there should be no logical reason for establishing a timewhen caries develop more generally than that at a certain period in a child's life he should have smallpox or asthma. There would seem to be more reason why children of the ages of, say two to six years, might be subjected to dental caries than at any other period; beause at this pèriod the hard dental tissues are in their more plastic and embryonic conditions and would necessarily be more liable to be infected or subject to traumatic influences.

Nature plans her growth, and changes take place with orderly regularity unless time and unusual environments decree otherwise. While we expect normal conditions to obtain in the development of the teeth, we know only too well that we have to assist nature by our artificial interference. As an illustration, the art of the orthodontist has given remarkable assistance to the best nature could do. We would not say that nature purposely or usually makes a miscarriage in dental embryology more often than she does in other byological parts of the system, but the frequency of failure in the hard tooth tissues lead one to suggest that there must be some cause for a tooth becoming carious at an earlier period than other conditions would warrant, the environmental cause alone.

Teeth are physically harder than other tissues and they logically should outlast other less resistent tissues, but they do not. Teeth are many times wholly destroyed within a short period of the time of their eruption, and outside of their environment their can be no reason for their being lost at untimely intervals. We think caries attack the teeth because they live in a specially destructive environment. We have hoped with our best endeavors to prevent decay by our artificial changes into more favorable environments, but all to no purpose so far. We can not believe that man is degenerating in spite of our more widespread scientific attainments. We yet expect to learn how to cure cancer, dental caries and other prevalent diseases.

For a long time we have had a notion we had not as yet fairly tested out a research that would determine whether children might not be given a prophylactic treatment at an early stage of their lives that would render their dental tissues immune to early carious destruction. People generally do not seem to understand that they need to care for their children's teeth, because the first teeth are lost early and it does not matter if the deciduous teeth do have to be extracted before the time for the permanent dentures to come in to replace them; also, because most parents think the surgeons and therapeutists will take care of the first teeth and no permanent loss can happen. But much is being accomplished by the orthodontists and dental hygienists and we believe that a new era of conservative dentistry is in prospect.

We are just now beginning to consider the bearing of food in its relation to better teeth. We have long believed that improper food, or a better adjustment of food values, especially for children, would play an important part in the future health of children, and especially with reference to preventive dentistry. This of course implies a prolonged and well worked out educational process, but it seems the only rational method of providing better dentistry if not better general health for the people. If there is any means that we have not as yet tried out, or if we have not as yet exhausted all our hygienic resources, let us try any new idea, or any old one which may be reworked successfully, if we can by such means stop the present hopeless and discouraging conditions that now face our profession. Is it practicable for us to start anew on a research that will give us a new inspiration and determination to make dentulous conditions practicable, and the rule, rather than that edentuolous oral tissues should prevail at the age of forty or fifty years?

We have had in recent years examples where some special need has been met by making a strenuous and concerted effort, and we have almost come to think we can do any difficult undertaking by enthusiastically getting together in a "*drive*," and the thing is done before we know it. Would it be practicable to enlist all the dentists of the country in a tremendous "*drive*" to take care of every child's teeth, by a proper hygienic or prophylactic treatment, until at least all the first set of teeth were safely preserved until the permanent set had begun to take their places. If this could be accomplished and it were demonstrated that by such care no children's first teeth would be lost or injured until the time has fully come for them to be replaced with the permanent successors, we believe there would be found enough dentists who would see to it that all teeth can and should be saved until age and disease removed them. This may seem so chimerical that a drive of this kind would hardly be practicable. Yet dreams more fanciful have materialized. If we can not organize a dental drive for the sake of our little children's teeth, can we not at least begin to think about how fine a thing it would be, and if we think hard enough about it, something like it will be sure to happen that will meet the need.

